# Supplementary material for: Hand2 delineates mesothelium progenitors and is reactivated in mesothelioma
Source: Nat Commun. 2022 Mar 30;13:1677. doi: 10.1038/s41467-022-29311-7 (PMC8967825; doi:10.1038/s41467-022-29311-7)
Supplement: Supplementary file 1 — Supplementary Information [file 41467_2022_29311_MOESM1_ESM.pdf]

Supplementary Information

Hand2 delineates mesothelium progenitors and is reactivated in mesothelioma

Prummel et al.

Supplementary Figures 1-9
Supplementary Table 1

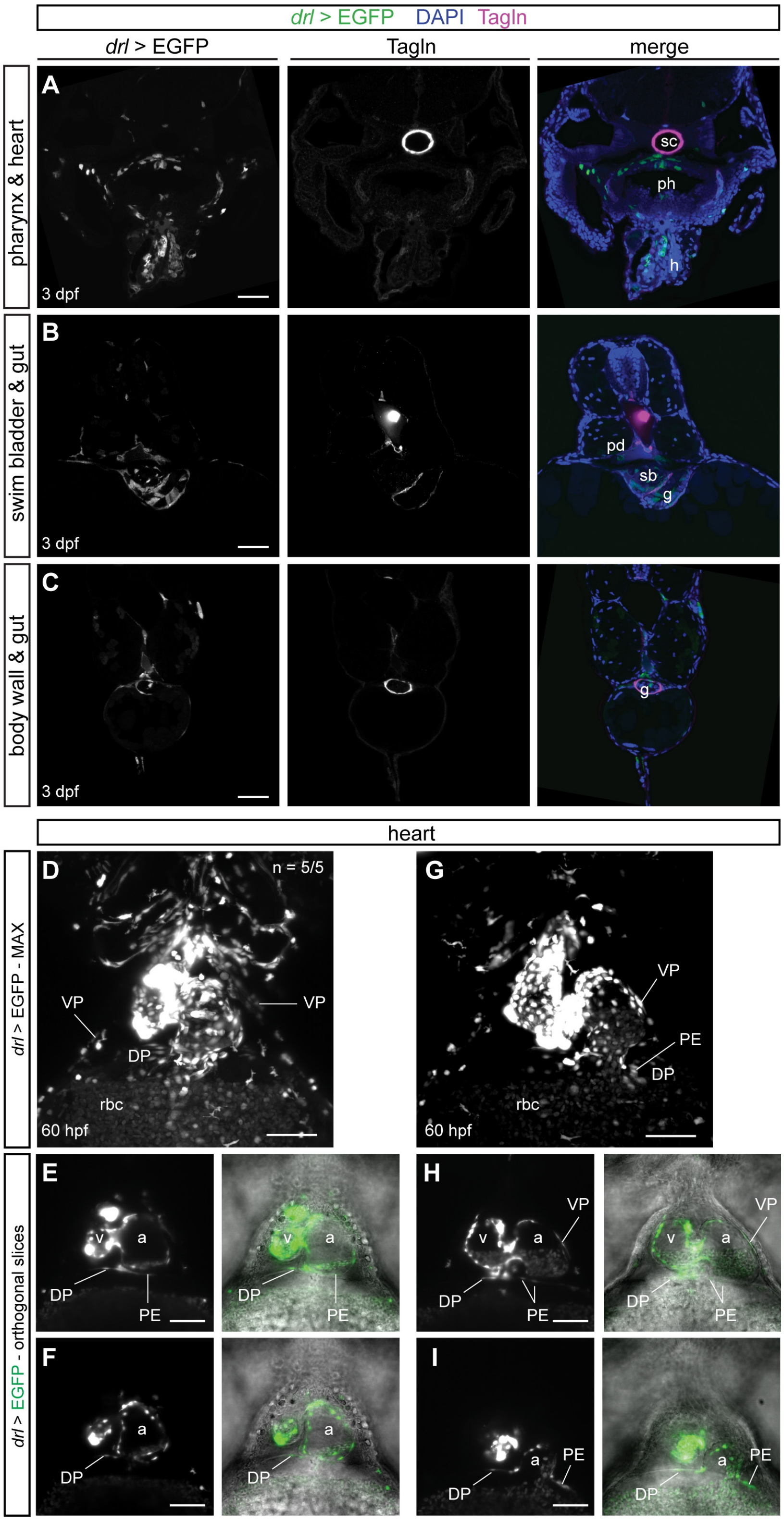

Supplementary Figure 1: *drl*-derived visceral and parietal mesothelial layers.
(A-C) Representative transverse sections of LPM lineage-traced *drl:creERT2*; hsp70l:Switch embryos at 3 dpf, co-stained with the smooth muscle marker Transgelin (Tagln). EGFP-expressed was observed in epithelial layers around the pharynx (**A**), swim bladder and gut (**B**), and as part of the body wall (parietal peritoneum) (**C**), in addition to other LPM-derived organs, including vasculature and the kidney tubules. **(D-I)** 4-OHT was administered at tailbud and washed off before 24 hpf. The embryos were heat-shocked at 60 hpf for 1 h and subsequently sorted for EGFP expression. (**D,G**) MAX projections of the heart of two representative *drl*-lineage traced embryos. Prior imaging, the heartbeat was stopped by administering 2,3-butanedione monoxime (BDM) to the embryo water. (**D,F**) EGFP expression in the ventral and dorsal pericardium. (**E,F,H,I**) show orthogonal slices along the Z-axis of the embryos corresponding to (**D**) and (**G**), revealing budding off proepicardial cells. Abbreviations: spinal cord (sc), heart (h), pharynx (ph), pronephric ducts (pd), swim bladder (sb), gut (g), ventral pericardium (VP), dorsal pericardium (DP), pro-epicardium (PE), epicardium (EP), red blood cells (rbc), ventricle (v), atrium (a). Scale bars (**A-C**) 50 µm and (**D-I**) 100 µm.

A

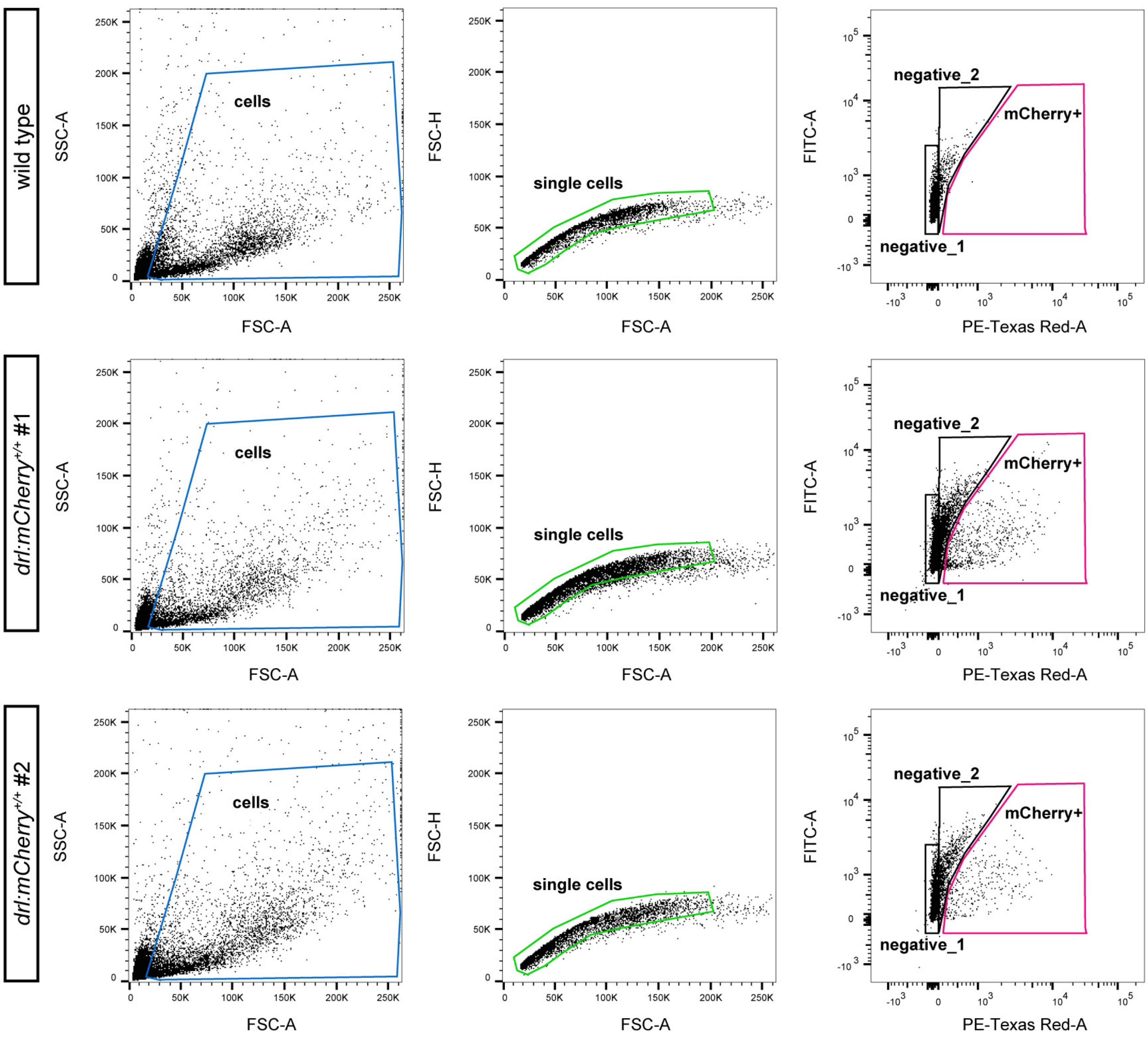

B

sample	total population		cells		single cells		mCherry+		negative_1		negative_2	
	# events	% parent	# events	% parent	# events	% parent	# events	% parent	# events	% parent	# events	% parent
wild type	10000	100	4428	44.3	4209	95.1	3	0.071	3842	91.3	411	9.8
drl:mCherry $^{+/+}$_1	100000	100	42515	42.5	38638	90.9	2517	6.51	27912	72.2	8373	21.7
drl:mCherry $^{+/+}$_2	11055	100	4626	41.8	4287	92.7	292	6.81	3036	70.8	962	22.4

Supplementary Figure 2: FACS of *drl:mCherry* embryos at tailbud stage.
(A) FACS plots show gating and sorting strategy for mCherry-positive cells within the single-cell suspension of *drl:mCherry*-dissociated embryos at tailbud stage. **(B)** Table shows the percentage of mCherry-positive cells in the total amount of cells.

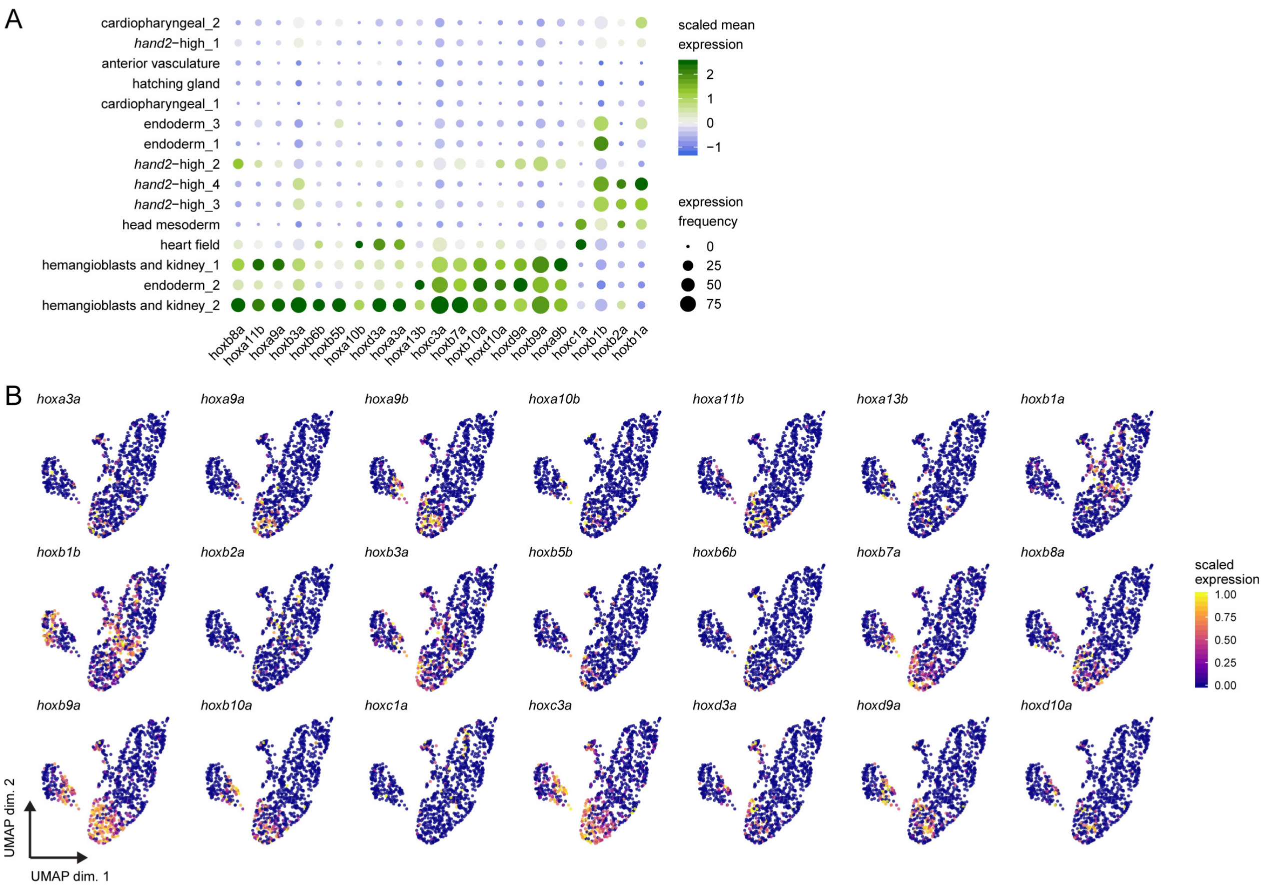

Supplementary Figure 3: *hox* gene expression across the LPM and endoderm at tailbud stage.
(A) Dotplot including all *hox* genes expressed throughout the 15 clusters. Dots are colored by column-scaled mean expression (log-transformed library-size-normalized counts), and sized by detection frequency (fraction of cells with non-zero counts); rows and clusters are ordered according to hierarchical clustering of scaled expression values. **(B)** UMAP plots of all the *hox* genes expressed throughout the data set. Cells are colored by scaled expression values using top and bottom 1%-quantiles as boundaries.

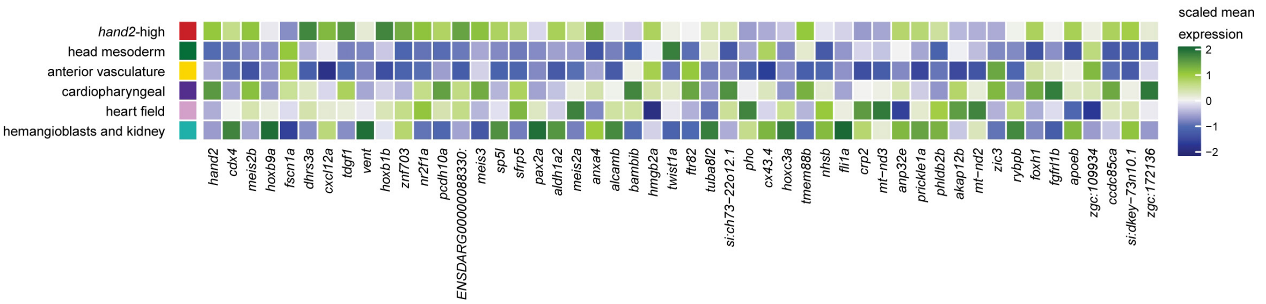

Supplementary Figure 4: predominant clusters and co-expressed genes within the LPM at tailbud stage. Heatmap of major subpopulation marker genes. Clusters with the same annotation (see **Fig. 2**) were manually merged into 6 'super' clusters: *hand2*-high, head mesoderm, anterior vasculature, cardiopharyngeal, heart field, and hemangioblasts and kidney. Displayed are the top 50 genes (in terms of effect size) that are differentially expressed against at least half of the remaining clusters (FDR < 5%, average log-fold change > 0); coloring corresponds to scaled mean expression.

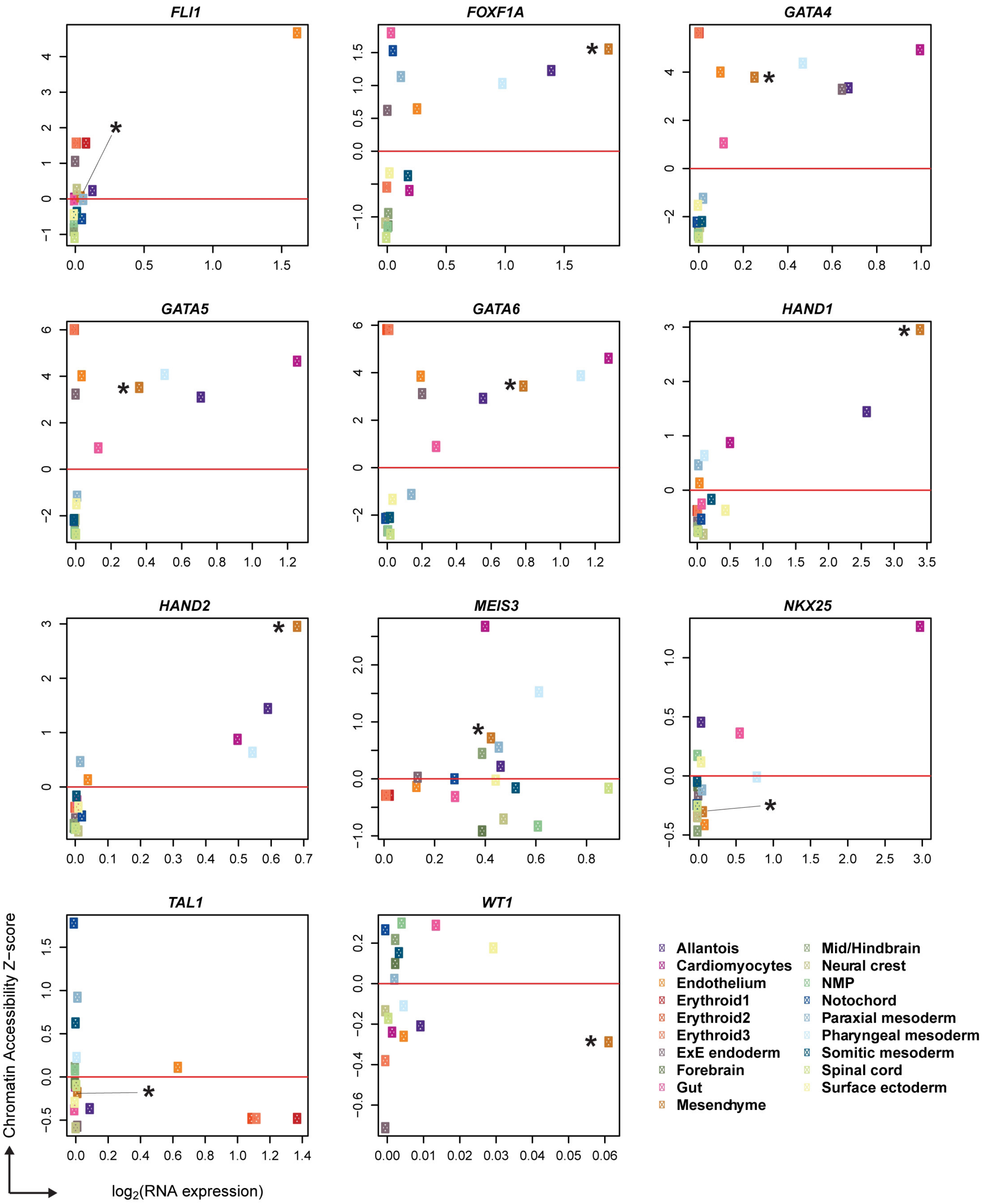

Supplementary Figure 5: mesothelial progenitors during mouse development as uncovered by co-expression of LPM genes.

Single-cell-based gene expression plots as published by Pijuan-Sala et al., 2020. Individual plots depict gene expression distribution of mouse orthologs of LPM-expressed genes, including genes found in zebrafish mesothelial progenitors. Color squares indicate individual organs and cell types, including mesenchyme (asterisks in individual plots). Mouse orthologs of mesothelial progenitor genes as defined in zebrafish including *Hand2*, *Hand1*, *GATA4/5/6*, *Meis3*, and *FoxF1* that is LPM-associated in tetrapods, show high relative expression in mesenchyme clusters, suggesting a mesothelial lineage identity.

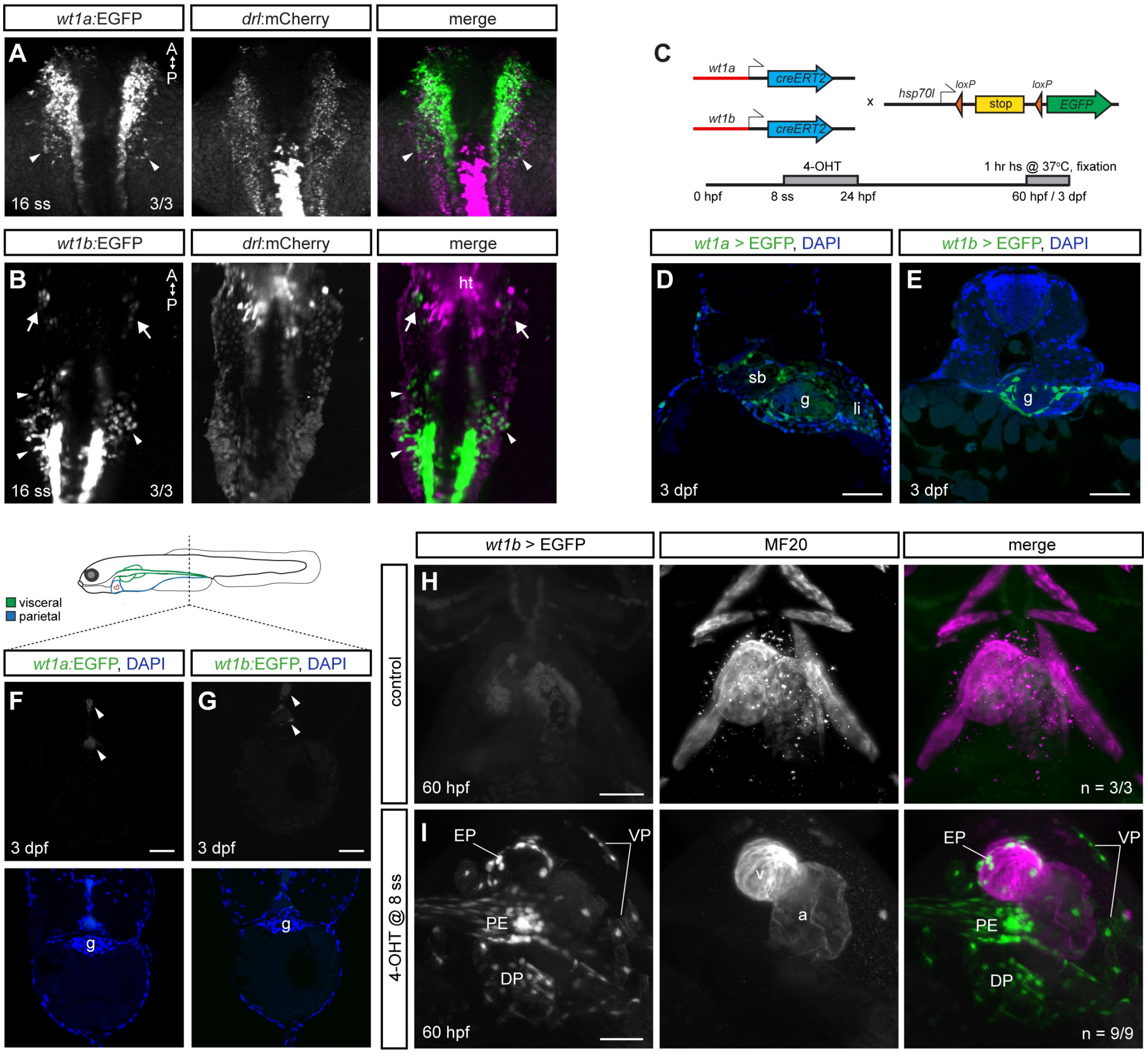

Supplementary Figure 6: *wt1a* and *wt1b* expression in the zebrafish LPM and in the peritoneal layers. (**A**) Confocal imaging of *wt1a:EGFP;drl:mCherry* embryos at 16 ss, showing EGFP-expression in the developing kidney and a more lateral LPM territory (arrowheads). (**B**) MAX Mercator projection of a *wt1b:EGFP;drl:mCherry* embryo at 16 ss. Note the cells lateral of the developing glomerulus (arrowheads) and in the pericardial progenitor field (arrows). (**C**) Tracing the fate of *wt1a- and wt1b*-derived cells using *wt1a:-* and *wt1b:creERT2* x *hsp70l:Switch*. 4-OHT administered at 8 ss and washed off before 24 hpf. *wt1a/-b>EGFP* indicates lineage labeling. (**D,E**) Transverse sections of *wt1a-* (**D**) and *wt1b*-lineage-traced embryos (**E**), demonstrating labeling of visceral peritoneum. (**F,G**) Posterior trunk transverse sections of *wt1a:-* (**F**) and *wt1b:EGFP*-expressing embryos (**G**), demonstrating absent labeling of the visceral peritoneum around the intestine in more posterior regions and absent labeling of the parietal peritoneum. (**H,I**) SPIM MAX of representative fixed control (n = 3/3) (**H**) and *wt1b*-lineage-traced embryos (n = 9/9) (I) stained with anti-Myosin heavy chain (MF20) in mCherry. Derivatives from *wt1b*-expressing cells are found in ventral and dorsal pericardium, pro-epicardium, and epicardium. Abbreviations: heart tube (ht), swim bladder (sb), gut (g), liver (li), ventral pericardium (VP), dorsal pericardium (DP), pro-epicardium (PE), epicardium (EP), ventricle (v), atrium (a). Nuclei in blue (DAPI). Scale bars (**D-G**) 50 µm and (**H,I**) 100 µm.

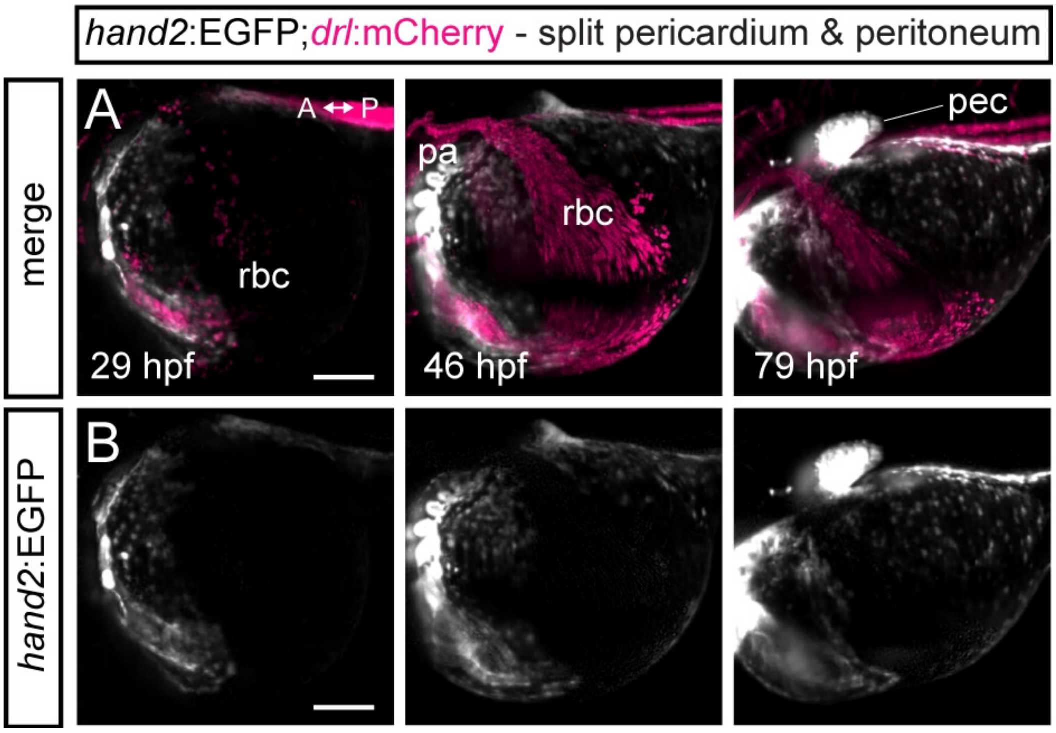

Supplementary Figure 7: blood flow over the yolk surface splits pericardium and parietal peritoneum. (**A**,**B**) Maximum intensity projections of the lateral angle of a *hand2:EGFP;drl:mCherry* double-transgenic zebrafish embryo at multiple time points. *drl:mCherry* (magenta in (**A**)) shows how the venous blood cells flow over the yolk surface (yolk circulation valley), boarder rostrally by forming pericardium and posterior by the forming parietal peritoneum. The yolk circulation valley is referred to as the duct of Cuvier when a vessel enclosing the blood cells is formed. Abbreviations: red blood cells (rbc), pharyngeal arches (pa), pectoral fin (pec). Scale bars 25 µm.

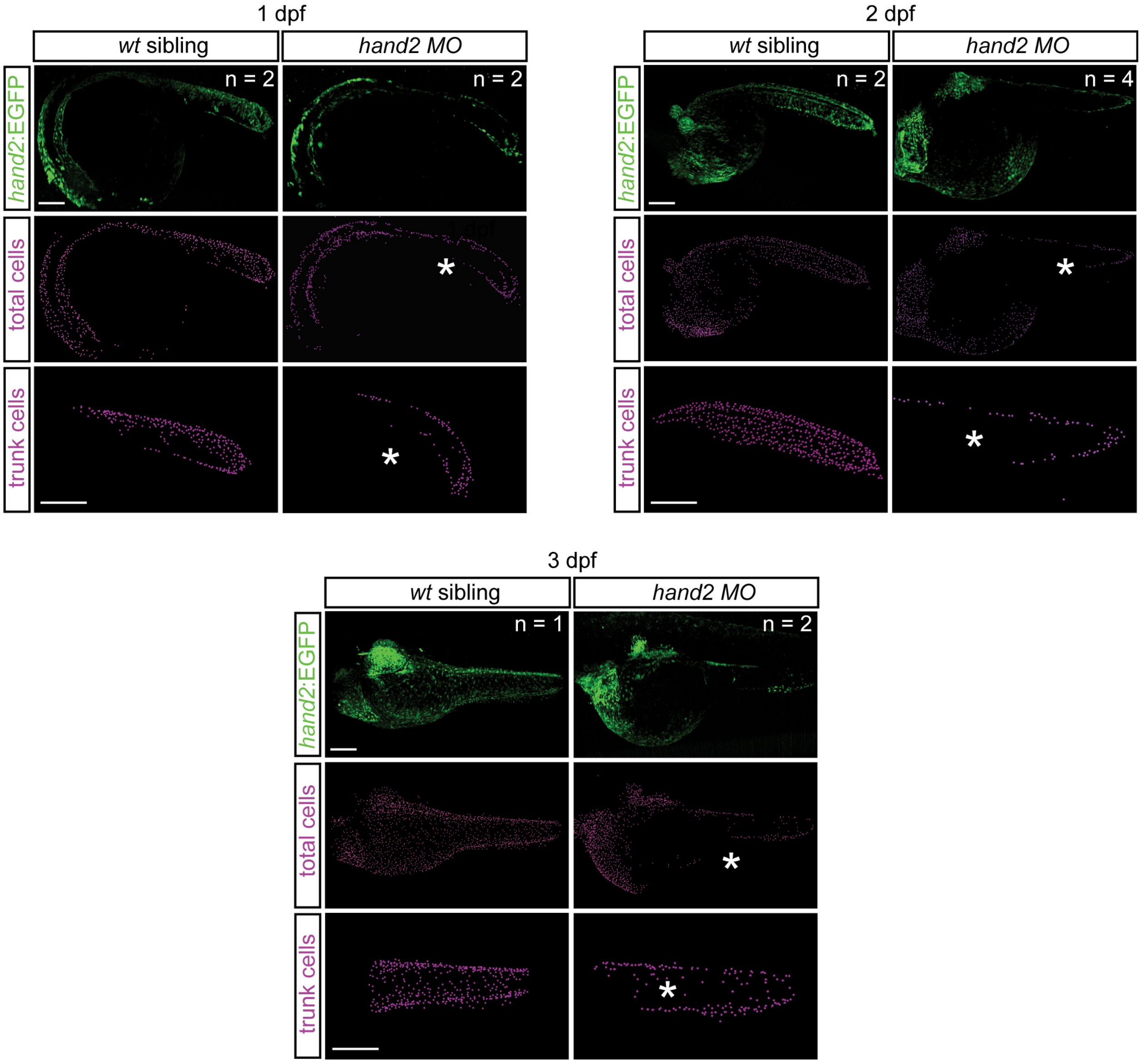

Supplementary Figure 8: imaging for quantification of *hand2*:EGFP-expressing cells in wildtype and upon *hand2* perturbation.
Light sheet-imaged *hand2*:EGFP-transgenic embryos, wildtype controls, and translation-blocking *hand2* morpholino-injected, imaged at 1, 2, and 3 dpf. Quantification of *hand2*:EGFP-expressing cells using Imaris is depicted for total cells as well as for trunk-only. Asterisks depict notable reduction of cell number and distribution over the yolk and yolk extension upon *hand2* perturbation. See main Figure 6 for numbers and additional phenotype description. Scale bars 100 µm.

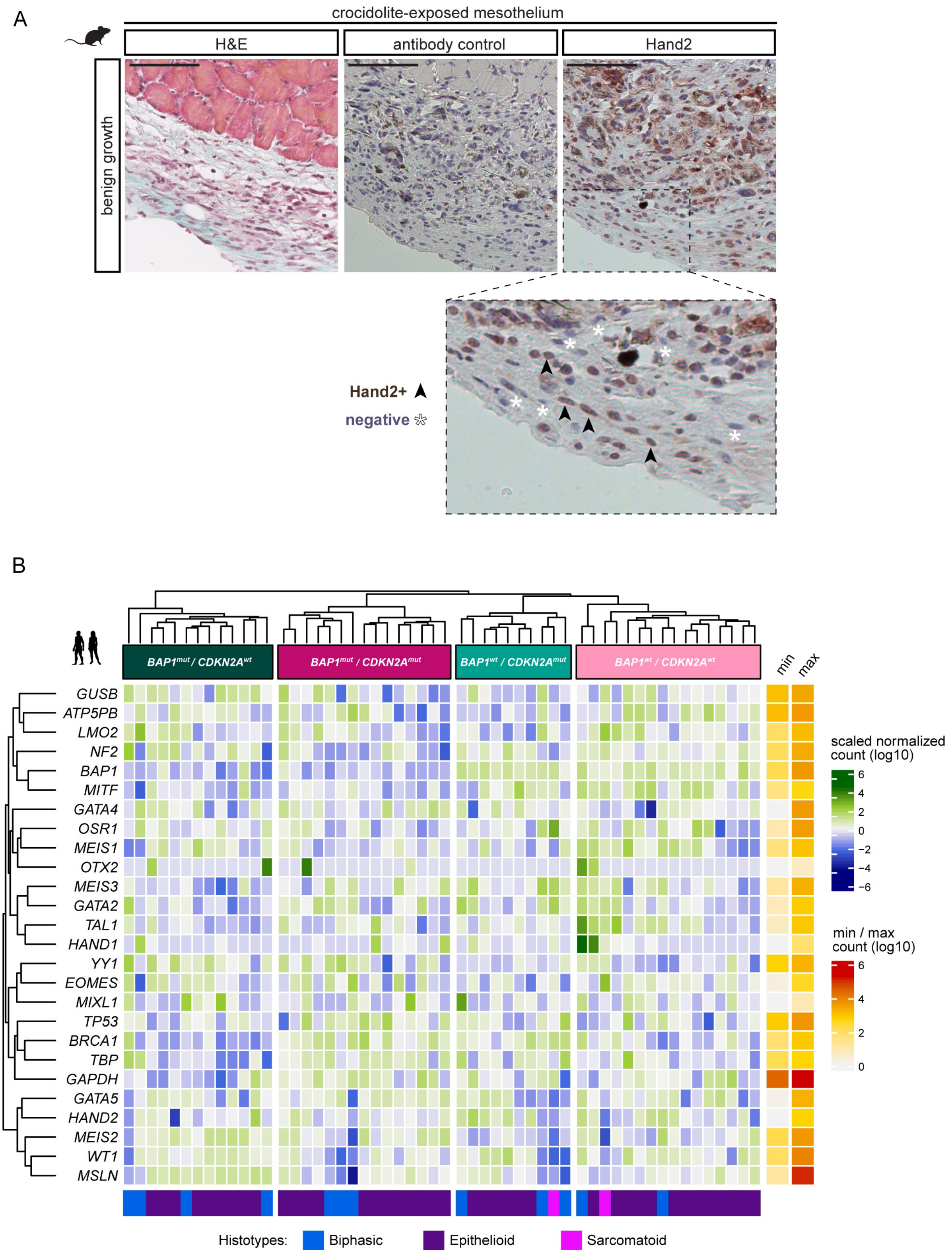

Supplementary Figure 9: re-activation of early LPM genes in mesothelioma in mouse and human.
(**A**) Sections of mouse mesothelium after crocidolite exposure, stained with H&E for tissue morphology, antibody staining control, and anti-Hand2 immunohistochemistry (brown staining). Magnification of anti-Hand2 staining shows blue unstained cells (white asterisks) and a brown fraction of Hand2-expressing cells (black arrowheads), indicative of induction of Hand2 in a fraction of mesothelial cells upon crocidolite exposure. Also compare to Figure 7. Scale bars 150 µm. (**B**) Mesothelial progenitor gene expression relative to BAP1 and CDKN2A mutation status in human mesothelioma. Heatmap representing hierarchical clustering of human mesothelial progenitor-associated LPM genes, mesothelioma-associated genes (*WT1*, *MSLN*, *BAP1*, *NF2*, *TP53*), unrelated control (*YY1*, *OTX2*, *BRCA1*), and ubiquitous housekeeping genes (*ATP5BP*, *GUSB*, *TBP*, *GAPDH*) across four different genomic TCGA malignant pleural mesothelioma (MPM) subtypes. TCGA MPM patients were stratified based on mutational profile of *BAP1* and *CDKN2A*. Columns represent MPM genomic subtypes and their corresponding histotypes, rows represent *log10*-transformed, batch-normalized mRNA expression levels of the indicated genes.

Tables

Supplementary Table 1:

Based on the genetic status of *BAP1* and *CDKN2A*, TCGA MPM (n = 54) patients were stratified into 4 subgroups. The mesothelioma patients with the following status were excluded from the analysis: i) wildtype *BAP1* but no *BAP1* expression, ii) *BAP1* with only one wildtype allele or *BAP1* of unknown status (*BAP1* is haploinsufficient). The genetic status of *NF2* is also indicated but not used for the subgrouping of the TCGA mesothelioma patients.
