# Supplementary material for: Hand2 delineates mesothelium progenitors and is reactivated in mesothelioma
Source: Nat Commun. 2022 Mar 30;13:1677. doi: 10.1038/s41467-022-29311-7 (PMC8967825; doi:10.1038/s41467-022-29311-7)
Supplement: Supplementary file 3 — Description of Additional Supplementary Files [file 41467_2022_29311_MOESM3_ESM.docx]

**Description of Additional Supplementary Files**

**Supplementary Movie 1:** Primordial germ cells are migrating within the drlpositive LPM during gastrulation and early somitogenesis Multiview time-lapse SPIM (Zeiss Z.1) of a representative embryo expressing drl:EGFP and primordial germ cell (PGC) marker nos-3’UTR:mCherry from 6 hpf until 14 hpf. Already during gastrulation, the PGCs migrate within the drl-expressing mesendoderm and concentrate within the LPM during early somitogenesis. Note the cells that end up more posterior in the most lateral territory of the drlpositive LPM.

**Supplementary Movie 2:** hand2 is expressed in the forming mesothelial layers Lateral view of dual-color timelapse imaging of a hand2:EGFP;drl:mCherry embryo from 18 hpf until 82 hpf. drl:mCherry drives expression in mainly the circulating blood cells from 24 hpf onwards. EGFP expression is restricted to the mesothelial layers, pectoral fin, pharyngeal arches, and heart (endo-, myocardium).

**Supplementary Movie 3:** hand2-expressing cells form the pericardium and visceral and parietal peritoneum Zebrafish embryo expressing hand2:EGFP imaged from four different angles over a time course from 18 hpf until 82 hpf. Full 3D-rendering shows how the EGFP-expressing cells migrate over the yolk and yolk extension to form the parietal peritoneum and the pericardium. A front of cells with a medial-directed migration pattern forms the visceral peritoneum along the anterior-to-posterior axis.

**Supplementary Movie 4:** hand2 mutants show anomalies in mesothelium migration SPIM-imaged 72 hpf (3 dpf) zebrafish embryos expressing hand2:EGFP in wildtype (left) and hanS6 homozygous mutant (right) background, anterior to the left, dorsal to the top at the beginning of the movie. Note the accumulating EGFP-expressing mesothelial progenitors that fail to cover the yolk (arrowheads in mutant, right). Embryos correspond to still images shown in Fig. 6.
