# Supplementary material for: Hand2 delineates mesothelium progenitors and is reactivated in mesothelioma
Source: Nat Commun. 2022 Mar 30;13:1677. doi: 10.1038/s41467-022-29311-7 (PMC8967825; doi:10.1038/s41467-022-29311-7)
Supplement: Supplementary file 9 — Reporting Summary [file 41467_2022_29311_MOESM9_ESM.pdf]

nature research

Corresponding author(s): Christian Mosimann

Last updated by author(s): Feb 18, 2022

Reporting Summary

Nature Research wishes to improve the reproducibility of the work that we publish. This form provides structure for consistency and transparency in reporting. For further information on Nature Research policies, see our Editorial Policies and the Editorial Policy Checklist.

Statistics

For all statistical analyses, confirm that the following items are present in the figure legend, table legend, main text, or Methods section.

n/a	Confirmed	
☐	☒	The exact sample size (*n*) for each experimental group/condition, given as a discrete number and unit of measurement
☐	☒	A statement on whether measurements were taken from distinct samples or whether the same sample was measured repeatedly
☐	☒	The statistical test(s) used AND whether they are one- or two-sided<br>*Only common tests should be described solely by name; describe more complex techniques in the Methods section.*
☐	☒	A description of all covariates tested
☐	☒	A description of any assumptions or corrections, such as tests of normality and adjustment for multiple comparisons
☐	☒	A full description of the statistical parameters including central tendency (e.g. means) or other basic estimates (e.g. regression coefficient) AND variation (e.g. standard deviation) or associated estimates of uncertainty (e.g. confidence intervals)
☐	☒	For null hypothesis testing, the test statistic (e.g. *F*, *t*, *r*) with confidence intervals, effect sizes, degrees of freedom and *P* value noted<br>*Give P values as exact values whenever suitable.*
☒	☐	For Bayesian analysis, information on the choice of priors and Markov chain Monte Carlo settings
☐	☒	For hierarchical and complex designs, identification of the appropriate level for tests and full reporting of outcomes
☒	☐	Estimates of effect sizes (e.g. Cohen's *d*, Pearson's *r*), indicating how they were calculated

Our web collection on statistics for biologists contains articles on many of the points above.

Software and code

Policy information about availability of computer code

Data collection	Primary data from developing zebrafish LPM was collected using scRNA-seq (Cel-Seq2) as outlined below and in the manuscript. All other sequencing data is derived from publicly available datasets.

Data analysis

All data analysis as outlined in the manuscript:

* Single-cell analysis
The CEL-Seq2 barcoded sequences were obtained from Single Cell Discoveries. STAR v2.5.3a (Dobin et al., 2013) was used to create an index based on the Ensembl GRCz10.91 genome and annotation, after manually adding drl:mCherry. Gene abundances were estimated separately for each plate using zUMIs v0.0.4 (Parekh et al., 2018), retaining cells with at least 100 reads and setting the Hamming distance threshold to 1 for both UMI and cell barcode identification. Finally, the exonic UMI counts for all four plates were merged into a single matrix.
Quality control and filtering were performed using the scater R package (McCarthy et al., 2017). Upon removal of genes that were undetected across all cells, we removed cells whose percentage of mitochondrial genes fell beyond 2 Median Absolute Deviations (MADs) of the median. Secondly, features with a count greater than 1 in at least 10 cells were retained for downstream analysis. Finally, we discarded cells measured on plate 4, as it was of overall low quality (low number of counts, high percentage of mitochondrial genes).
Next, we used Seurat (Stuart et al., 2019) for clustering and dimension reduction. Clustering was performed using the 2000 most highly variable genes (HVGs) identified via Seurat's FindVariableFeatures function with default parameters; clustering and dimension reductions were computed using the first 20 principal components. For clustering, we considered a range of resolution parameters (0.2-2.4); downstream analyses were performed on cluster assignments obtained from resolution 1.8 (15 subpopulations).
Cluster annotations were performed manually on the basis of canonical markers in conjunction with marker genes identified programmatically with Seurat's FindAllMarkers function, and complementary exploration with iSEE (Rue-Albrecht et al., 2018).
For Supplementary Figure 4, the three endoderm clusters were excluded and the remaining clusters manually merged into 6 major subpopulations. For each subpopulation, genes that were differentially expressed (DE) against at least 3 others were identified using scater's findMarkers function with pval.type = "some" (McCarthy et al., 2017). The top 50 DE genes (in terms of effect size) at FDR < 5% and with an

average positive log-fold change (summary.logFC > 0) were selected for visualization.

All analyses were run in R v4.0.2, with Bioconductor v3.11. Data preprocessing and analysis code are deposited at DOI:10.5281/zenodo.4267898, and available as a browsable workflowr website (Blischak et al., 2019). All package versions used throughout this study are captured in the session info provided therein. Package details and versions:
scran_1.16.0
scater_1.16.2
ggplot2_3.3.2
SingleCellExperiment_1.10.1
SummarizedExperiment_1.18.2
DelayedArray_0.14.1
matrixStats_0.57.0
Biobase_2.48.0
GenomicRanges_1.40.0
GenomeInfoDb_1.24.2
IRanges_2.22.2
S4Vectors_0.26.1
BiocGenerics_0.34.0
dplyr_1.0.2
biomaRt_2.44.4
workflowr_1.6.2

* Mouse RNA-seq analysis
The RNA isolation, library generation, and RNA-seq analysis pipelines are previously described (Rehrauer et al., 2018). RNA-seq data from this previous study was used as deposited at the European Nucleotide Archive (ENA) under project accession PRJEB15230 (http://www.ebi.ac.uk/ena/data/view/PRJEB15230). Here, we extracted the expression values of genes associated with early LPM and mesothelium development and diagnostic markers (Msln, Wt1, Bap1, Nf2). Counts were normalized using size factors calculated with DESeq2's estimateSizeFactors function (Love et al., 2014); heatmap values correspond to log10-transformed, scaled and centered (scale with default parameters) normalized counts. Right-hand side row annotations display (unscaled) log10-transformed count ranges.

* Human RNA-seq analysis
To perform unsupervised clustering analysis on the mRNA of TCGA mesothelioma samples, we used the complexHeatmap R package (Gu et al., 2016). Heatmap values correspond to log10-transformed, batch-normalized mRNA counts. The human mesothelioma RNA-seq analyses were run in R v4.1.0. Call code is deposited at DOI:10.5281/zenodo.5879980.

* Image Analysis:
Image conversion, LUT conversions, and basic manipulations were performed using Fiji*ImageJ 1.52q 13 (Java 8).
SPIM images were converted and analyzed using Imaris 9.5, 9.6, and 9.7
2D projections were performed using Matlab 9.7 with the scripts deposited here: https://github.com/sundar07/Mesothelium

*Cell sorting
Data was handled using FlowJo 10.2.

* Plots
Plots for cell number quantification were performed using GraphPad Prism (9.3.0.).

For manuscripts utilizing custom algorithms or software that are central to the research but not yet described in published literature, software must be made available to editors and reviewers. We strongly encourage code deposition in a community repository (e.g. GitHub). See the Nature Research guidelines for submitting code & software for further information.

Data

Policy information about availability of data

All manuscripts must include a data availability statement. This statement should provide the following information, where applicable:
- Accession codes, unique identifiers, or web links for publicly available datasets
- A list of figures that have associated raw data
- A description of any restrictions on data availability

- All new sequencing datasets generated for this publication are deposited on the ArrayExpress database as E-MTAB-9727 (http://www.ebi.ac.uk/arrayexpress/experiments/E-MTAB-9727); raw scRNA-seq data, intermediate, and metadata files to reproduce all analyses and figures are available at DOI:10.6084/m9.figshare.13221053.v1.
- Analysis of human mesothelioma was performed using TCGA data downloaded from cBioportal (www.cBioportal.org) (Gao et al., 2013) and all code used is deposited at DOI:10.5281/zenodo.5879980.
- MATLAB image processing codes are available at https://github.com/sundar07/Mesothelium.
- All reagents used are freely available upon request.

Field-specific reporting

Please select the one below that is the best fit for your research. If you are not sure, read the appropriate sections before making your selection.

☒ Life sciences ☐ Behavioural & social sciences ☐ Ecological, evolutionary & environmental sciences

For a reference copy of the document with all sections, see nature.com/documents/nr-reporting-summary-flat.pdf

Life sciences study design

All studies must disclose on these points even when the disclosure is negative.

Sample size	All experimental sample sizes were chosen by common standards in the field and in accordance with solid phenotype designation; mutants were analyzed to reach an n larger than 3 as outlined in the manuscript, using replicate clutches from independent zebrafish matings to ensure robustness of the resulting analyses for hand2 mutant phenotypes (Yelon et al., 2000); scRNA-seq data was validated separately by statistical means as outlined above and in the manuscript; human and mouse tumor data was analyzed based on available sample size (Rehraurer et al., 2018).
Data exclusions	Supplementary Table 1:<br>Based on the genetic status of BAP1 and CDKN2A, TCGA MPM (n=54) patients were stratified into 4 subgroups. The mesothelioma patients with the following status were excluded from the analysis: i) wildtype BAP1 but no BAP1 expression, ii) BAP1 with only one wildtype allele or BAP1 of unknown status (BAP1 is haploinsufficient). The genetic status of NF2 is also indicated but not used for the subgrouping of the TCGA mesothelioma patients.
Replication	Imaging and mutant analysis experiments were performed at least twice and three or more times in the majority of instances. All attempts at replication were successful and validated further by additional experiments, such as validation of gene expression by other means (see i.e. Figure 3).
Randomization	Zebrafish embryos for scRNA-seq were by the very nature of zebrafish embryo collection randomized. Data analyses for mutant quantification was based on defined genotypes and injections arising from the obtained zebrafish crosses. No other randomizations are applicable.
Blinding	Data collection for mutant analyses was unblinded as it required phenotype assessment and genotyping analysis to confirm mutant versus wildtype. All data analysis of mutant phenotypes was confirmed using blinded image names and co-authors not involved in the primary data collection.

Reporting for specific materials, systems and methods

We require information from authors about some types of materials, experimental systems and methods used in many studies. Here, indicate whether each material, system or method listed is relevant to your study. If you are not sure if a list item applies to your research, read the appropriate section before selecting a response.

Materials & experimental systems

n/a	Involved in the study	
☐	☒	Antibodies
☒	☐	Eukaryotic cell lines
☒	☐	Palaeontology and archaeology
☐	☒	Animals and other organisms
☐	☒	Human research participants
☒	☐	Clinical data
☒	☐	Dual use research of concern

Methods

n/a	Involved in the study	
☒	☐	ChIP-seq
☐	☒	Flow cytometry
☒	☐	MRI-based neuroimaging

Antibodies

Antibodies used	anti-Pax2 (GeneTex, GTX128127, 1:250), anti-Myosin heavy chain (DSHB, MF20, 1:20), and anti-HAND2 antibody (Santa Cruz, Sc-398167, 1:100), and anti-SM22 (Transgelin) (AbCam, ab14106, 1:250).<br>Secondary antibodies used were goat-anti-rabbit Alexa Fluor 594 (Life Technologies, A-11012, 1:300), goat-anti-rabbit Alexa633 (Alexa Fluor, Life Technologies, A-21070, 1:300-400), goat-anti-mouse Alexa594 (Alexa Fluor, Life Technologies, A-11032, 1:300-400), and a universal secondary staining antibody cocktail (Vectastain kit, Vector laboratories, PK-6200, prepared as per manufacturer's manual and kit reagents using 100 μL blocking serum stock in 5 ml buffer with additional 100 μL of secondary antibody stock as working solution).
Validation	- anti-Pax2 GTX128127 has been validated on zebrafish whole-mount stainings and comparison to the known pax2a gene expression pattern by GeneTex (https://www.genetex.com/Product/Detail/Pax2a-antibody/GTX128127).<br>- MF20 recognizes MYH1E and is a long-standing standard reagent in the field, with validation/characterization reported here: https://doi.org/10.1007/978-3-319-93560-7_9.

- Sc-398167, has been validated using Western Blot and immunohistochemistry: https://www.scbt.com/p/dhand-antibody-a-12.
- ab14106 has been validated using Western blot and immunohistochemistry: https://www.abcam.com/taglntransgelin-antibody-ab14106.html

Animals and other organisms

Policy information about studies involving animals; ARRIVE guidelines recommended for reporting animal research

Laboratory animals	Zebrafish; AB, TU wildtypes; embryos were not selected by sex as determination happens later in development.
Wild animals	No wild animals were used in the study.
Field-collected samples	No field collected samples were used in the study.
Ethics oversight	The study was approved by the IACUC at the University of Colorado School of Medicine, Anschutz Medical Campus (protocol no. 00370); the cantonal veterinary office of the Canton Zurich (Kantonales Veterinäramt, permit no. 150, TV4209); and the Institutional Ethical Review Board of the University Hospital Zurich under reference number StV 29-2009.

Note that full information on the approval of the study protocol must also be provided in the manuscript.

Human research participants

Policy information about studies involving human research participants

Population characteristics	Mesothelioma tumor specimens were collected from MPM patients with patient consent, as treated at the Department of Medical Oncology and Department of Thoracic Surgery between January 2007 and December 2014. Non-tumor pleural tissue was received with consent from four patients undergoing mesothelioma-unrelated thoracic surgery.
Recruitment	All participants were patients of the Department of Medical Oncology and Department of Thoracic Surgery, University Hospital Zurich. All participants consented to the use of their tissue samples and to participation in the research study.
Ethics oversight	The Zurich Cantonal Ethics Committee approved the study under the reference number StV 24-2005 and 29-2009, and informed consent was collected from all patients.

Note that full information on the approval of the study protocol must also be provided in the manuscript.

Flow Cytometry

Plots

Confirm that:

- [x] The axis labels state the marker and fluorochrome used (e.g. CD4-FITC).
- [x] The axis scales are clearly visible. Include numbers along axes only for bottom left plot of group (a 'group' is an analysis of identical markers).
- [x] All plots are contour plots with outliers or pseudocolor plots.
- [x] A numerical value for number of cells or percentage (with statistics) is provided.

Methodology

Sample preparation	All FACS was performed for scRNA-seq sample isolation and not for biological analysis per se.<br><br>drl:mCherry-positive cells were sorted using a FACS Aria III cell sorter (BD Bioscience). Cells were gated based on size and forward scattering, to exclude debris and doublets. The gating for the negative population was determined based on wildtype tailbud stage embryos. See also Supplementary Figure 2 for gate setting. The SORTseq single-cell RNA-sequencing protocol was carried out as described previously (Muraro et al., 2016). Live mCherry-positive single cells were sorted in four 384-well plates (BioRad) containing 5 µl of CEL-Seq2 primer solution in mineral oil (24 bp polyT stretch, a 4 bp random molecular barcode (UMI), a cell-specific barcode, the 5'Illumina TruSeq small RNA kit adaptor and a T7 promoter), provided by Single Cell Discoveries. After sorting, the plates were immediately placed on ice and stored at −80°C.
Instrument	FACS Aria III cell sorter (BD Bioscience)
Software	FlowJo, FACS Aria III cell sorter operation software 10.2
Cell population abundance	drl:mCherry-positive cells made up approximately 7% of the entire population, and we in the end processed about 1k cells for scRNA-seq.
Gating strategy	The gating for the negative population was determined based on wildtype tailbud stage embryos. See also Supplementary Figure 2 for gate setting.

- [x] Tick this box to confirm that a figure exemplifying the gating strategy is provided in the Supplementary Information.
